# Challenges and care strategies associated with the admission to nursing homes in Germany: a scoping review

**DOI:** 10.1186/s12912-022-01139-y

**Published:** 2023-01-05

**Authors:** Stefanie Skudlik, Julian Hirt, Tobias Döringer, Regina Thalhammer, Katharina Lüftl, Birgit Prodinger, Martin Müller

**Affiliations:** 1 Centre for Research, Development and Technology Transfer, Rosenheim Technical University of Applied Sciences, Rosenheim, Germany; 2 International Graduate Academy, Medical Faculty, Institute for Health and Nursing Science, Martin Luther University Halle-Wittenberg, Halle (Saale), Germany; 3 Institute for Applied Nursing Science, Department of Health, Eastern Switzerland University of Applied Sciences (Formerly FHS St. Gallen), St. Gallen, Switzerland; 4 Department of Clinical Research, University Hospital Basel, University of Basel, Basel, Switzerland; 5 Faculty of Applied Health and Social Sciences, Rosenheim Technical University of Applied Sciences, Rosenheim, Germany; 6 Department for Primary Care and Health Services Research, Medical Faculty, Nursing Science and Interprofessional Care, Heidelberg University, Heidelberg, Germany

**Keywords:** Nursing home, Admission, Challenges, Care strategies, Participation, Older adults, Scoping review

## Abstract

**Background:**

The admission to a nursing home is a critical life-event for affected persons as well as their families. Admission related processes are lacking adequate participation of older people and their families. To improve transitions to nursing homes, context- and country-specific knowledge about the current practice is needed. Hence, our aim was to summarize available evidence on challenges and care strategies associated with the admission to nursing homes in Germany.

**Methods:**

We conducted a scoping review and searched eight major international and German-specific electronic databases for journal articles and grey literature published in German or English language since 1995. Further inclusion criteria were focus on challenges or care strategies in the context of nursing home admissions of older persons and comprehensive and replicable information on methods and results. Posters, only-abstract publications and articles dealing with mixed populations including younger adults were excluded. Challenges and care strategies were identified and analysed by structured content analysis using the TRANSCIT model.

**Results:**

Twelve studies of 1,384 records were finally included. Among those, seven were qualitative studies, three quantitative observational studies and two mixed methods studies. As major challenges neglected participation of older people, psychosocial burden among family caregivers, inadequate professional cooperation and a lack of shared decision-making and evidence-based practice were identified. Identified care strategies included strengthening shared decision-making and evidence-based practice, improvement in professional cooperation, introduction of specialized transitional care staff and enabling participation for older people.

**Conclusion:**

Although the process of nursing home admission is considered challenging and tends to neglect the needs of older people, little research is available for the German health care system. The perspective of the older people seems to be underrepresented, as most of the studies focused on caregivers and health professionals. Reported care strategies addressed important challenges, however, these were not developed and evaluated in a comprehensive and systematic way. Future research is needed to examine perspectives of all the involved groups to gain a comprehensive picture of the needs and challenges. Interventions based on existing care strategies should be systematically developed and evaluated to provide the basis of adequate support for older persons and their informal caregivers.

**Supplementary Information:**

The online version contains supplementary material available at 10.1186/s12912-022-01139-y.

## Background

### Care-dependency in Germany and increase in nursing home admissions

In Germany, the risk of developing the need for long-term nursing care rises with age and is nearly up to 50% in people aged 85 years and older [1]. The majority of people with the need for long-term nursing care in Germany are living at home. About 20% live in long-term care facilities [1]. It is projected that there will be a decline of the potential of informal care giving, mainly due to the increase in the prevalence of multimorbidity, hence more complex care needs [2, 3], and changes to social structures such as demographic prognosis, an expected sharp increase in the number of care-dependent people [4] and the spatial separation of families [5]. Although the majority of people prefer to receive long-term care in their own homes for as long as possible [6–8], the expected decline in informal care potential could lead to an increase of admissions to long-term care facilities.. Health care in Germany is provided by the statutory health insurance for acute illness and the long-term care insurance. Both are part of the mandatory social insurance system. Nursing homes are either run by communities, welfare or private organizations and are financed by the German statutory long-term care insurance supplemented by residents’ payments.

### Challenges of nursing home admissions for individuals

The admission to a nursing home is a critical life event [9, 10] Research has shown an association with psycho-social burden for both people in need of care and their informal caregivers [9, 11–13]. Older people may experience a decrease in social participation and restrictions in daily routines and autonomy [9, 14–16] which can result in feelings of loss of identity [17], loneliness, anxiety and depression [9, 18, 19]. Informal caregivers face different emotional strains such as feelings of shame, self-blame, loneliness and grief [20]. Negative experiences like insufficient preparation for the nursing home admission [20–22] including lack of support from health professionals (HPs) and lack of inclusion in the decision-making-process [22], and also fragmented transitional care [23, 24], can even worsen these circumstances.

### Challenges of nursing home admissions for the German health system

In order to adequately respond to the challenging situation of nursing home admission, affected individuals and their informal caregivers need support from the health care system and the different health professions involved. There are approaches to improve the quality of transitional situations in Germany such as the national experts’ standard for hospital discharge management [25]. However, transitional processes still pose a risk to the safety of the people in need of care [26]. Approaches from health care providers are often inadequate in addressing the complexity of the situation, with a lack of inclusion of the affected individuals and informal caregivers in decision-making processes [27]. Even though there are programmes from other countries available, many seem to have inconsistent intervention components and results, or have not been systematically evaluated [28, 29] and therefore can’t provide sufficient guidance. In addition, programmes cannot be easily transferred to other health care health system due to the different contextual factors which have crucial influence on the implementation strategies and the success of the intervention [28, 29], such as education, staffing requirements, reimbursement, or interprofessional collaboration [30].

Moreover, admissions from different settings may require different approaches for a successful admission management. A representative survey among German nursing homesreported that most nursing home admissions (59%) took place from home to nursing home, followed by admissions from acute care hospitals (24%), rehabilitation facilities (6%) and mental health facilities (5%) [31].

Comprehensive knowledge about the challenges of admissions to nursing homes and successful approaches to address these challenges can help design new comprehensive interventions to enhance participation, quality of life and quality of care. Therefore, the aim of this review was to identify the available evidence regarding challenges of nursing home admissions and care strategies in Germany.

The aim was to address the following research questions:

1. What are the challenges encountered by people in need of nursing care, by their informal caregivers, and by healthcare professionals and providers associated with nursing home admissions?
2. What are the approaches and care strategies addressing the challenges of nursing home admissions in Germany?

## Methods

We decided to conduct a scoping review in order to address the research questions. Scoping reviews are an appropriate way to identify research gaps, to make recommendations for further research, to determine the range of available evidence and finally to bundle and communicate research results [32]. Another reason for the decision was that scoping reviews allow the inclusion of all levels and types of evidence. A protocol for the scoping review is available at OpenScienceFramework [33].

We adhered to the methodology for Joanna Briggs Institute (JBI) scoping reviews [32] and to the Preferred Reporting Items for Systematic Reviews and Meta-Analyses extension for Scoping Reviews (PRISMA-ScR) [34]. The completed PRISMA-ScR checklist can be found in additional file 1.

### Eligibility criteria

Studies were considered eligible if the criteria in Table 1 were met.

**Table 1:** Eligibility criteria

Key elements	Eligibility criteria
Population	**(1)** Older individuals (aged 65 or older) who are in need of nursing care and were admitted to nursing homes **(2)** Health professionals/ health providers or informal caregivers (families/friends, paid/unpaid) who were involved in the admission to the nursing home
Concept	Challenges and care strategies (e.g., interventions, best-practice examples, recommendations) of admissions to nursing homes
Context	**(1)** Admissions to nursing homes in Germany (this setting includes discharging settings, e.g., acute care hospitals, rehabilitation facilities, and other nursing homes as discharging institutions) **(2)** Publication languages*:* German, English **(3)** Publication date*:* since 1995 (introduction of the Social Security Code XI (SGB XI), the German long-term care insurance) **(4)** Types of evidence sources: all study types with an IMRaD structure (Introduction, Materials and Methods, Results, Discussion and Conclusions) including (i) peer-reviewed and non-peer-reviewed journal articles and conference proceedings, (ii) grey literature such as preprints and reports from official agencies/policy documents, and doctoral theses **Excluded evidence sources:** (i) Poster and only-abstract publications; (ii) articles dealing with mixed populations including younger adults

### Information sources

We searched Web of Science Core Collection, CareLit, CINAHL, MEDLINE via PubMed, CC Med and PSYNDEX via LIVIVO, PROSPERO and Google Scholar, supplemented by web-searching via Google.

### Search

In accordance with the JBI methodology [32], the development of the search strategy consisted of the following steps: 1) A limited search in the Web of Science Core Collection to identify keywords and index terms and a thesaurus search and brainstorming in the working group, 2) a search of all relevant data sources using the identified keywords and index terms, and 3) screening the existing reference list for additional studies. The list was supplemented by web-searching via Google.

The search strategy was developed by one reviewer (StS) and reviewed by a second (JH) by using the evidence-based guideline for Peer Review of Electronic Search Strategies (PRESS) [35]. The search was performed on 23^rd^ June 2021.

We used a sensitive search strategy which includes the terms ‘nursing home’, ‘admission’ and ‘Germany’. The final database-specific search strategies are given in Table 2.

**Table 2:** Search strategy 23.06.2021

MEDLINE via Pubmed			
**#**	**Input**		**Hits**
**#**	"NURSING HOME*"[TITLE/ABSTRACT] OR "LONG-TERM CARE"[TITLE/ABSTRACT] OR "NURSING HOMES"[MESH TERMS]		67,867
**#**	"ENTRY"[TITLE/ABSTRACT] OR "TRANSITION*"[TITLE/ABSTRACT] OR "PLACEMENT"[TITLE/ABSTRACT] OR "ADMISSION*"[TITLE/ABSTRACT] OR "DISCHARG*"[TITLE/ABSTRACT] OR "PATIENT DISCHARGE"[MESH TERMS] OR "PATIENT TRANSFER"[MESH TERMS] OR "TRANSITIONAL CARE"[MESH TERMS] OR "PATIENT ADMISSION"[MESH TERMS]		1,198,833
**#**	"GERMAN*"[TITLE/ABSTRACT] OR "GERMAN*"[AFFILIATION] OR "DEUTSCH*"[AFFILIATION] OR "GERMANY"[MESH TERMS]		1,275,601
**#**	#1 AND #2 AND #3		428
**#**	#4 FILTERS APPLIED: FROM 1995—3000/12/12		403
**Web of Science Core Collection**			
**#**	**Input**		**Hits**
**1**	TS = ("NURSING HOME*" OR "LONG-TERM CARE") INDEXES = SCI-EXPANDED, SSCI, A&HCI, CPCI-S, CPCI-SSH, ESCI TIMESPAN = 1995–2021		51,298
**2**	TS = (ENTRY OR TRANSITION* OR PLACEMENT OR ADMISSION* OR DISCHARG*) *INDEXES* = *SCI-EXPANDED, SSCI, A&HCI, CPCI-S, CPCI-SSH, ESCI TIMESPAN* = *1995–2021*		2,686,846
**3**	TS = (GERMAN* OR DEUTSCH*) OR OO = ( GERMAN* OR DEUTSCH*) OR CU = GERMANY *INDEXES* = *SCI-EXPANDED, SSCI, A&HCI, CPCI-S, CPCI-SSH, ESCI TIMESPAN* = *1995–2021*		3,608,966
**4**	#3 AND #2 AND #1 *INDEXES* = *SCI-EXPANDED, SSCI, A&HCI, CPCI-S, CPCI-SSH, ESCI TIMESPAN* = *1995–2021*		538
**CINAHL**			
**#**	**Input**		**Hits**
**#**	TI "NURSING HOME*" OR AB "NURSING HOME*" OR TI "LONG-TERM CARE" OR AB "LONG-TERM CARE" OR MH NURSING HOMES		49,065
**#**	TI ENTRY OR AB ENTRY OR TI TRANSITION* OR AB TRANSITION* OR TI PLACEMENT OR AB PLACEMENT OR TI ADMISSION* OR AB ADMISSION* OR TI DISCHARG* OR AB DISCHARG* OR MH TRANSITIONAL CARE OR MH PATIENT ADMISSION OR MH PATIENT DISCHARGE +		284,755
**#**	TI GERMAN* OR AB GERMAN* OR TI DEUTSCH* OR AB DEUTSCH* OR AF GERMAN* OR AF DEUTSCH* OR MH GERMANY		177,279
**#**	#1 AND #2 AND #3		193
**#**	LIMITERS—PUBLISHED DATE: 19,950,101–20,211,231		192
**CC Med and PSYNDEX via LIVIVO**			
**#**	**Input**		**Hits**
**#**	FS = ("NURSING HOME" OR "NURSING HOMES" OR "LONG-TERM CARE" OR ALTENHEIM* OR ALTENHEIME OR PFLEGEHEIM* OR PFLEGEHEIME OR LANGZEITPFLEGE)		9965
**#**	FS = (ENTRY OR TRANSITIONS* OR TRANSITION OR PLACEMENT OR ADMISSION* OR ADMISSION OR DISCHARG* OR DISCHARGE OR EINZUG OR UMZUG OR ÜBERGANG OR EINTRITT OR ENTLASSUNG OR ÜBERLEITUNG)		17,148
**#**	FS = (GERMAN* OR DEUTSCH*)		214,258
**#**	#1 AND #2 AND #3		46
**#**	FILTER AB 1995		34
**PROSPERO**			
**#**	Input		**Hits**
**#**	(NURSING HOME* OR LONG-TERM CARE)		1933
**#**	(ENTRY OR TRANSITION* OR PLACEMENT OR ADMISSION* OR DISCHARG*)		13,528
**#**	#1 AND #2		557
**Google Scholar**			
**#**	**Input**		**Hits**
**#**	NURSING HOME ADMISSION GERMANY		Ca. 385.000
**#**	PFLEGEHEIM EINTRITT		Ca. 9.830
**CareLit**			
**#**	**Input**		**Hits**
**#**	TITEL = ALTENHEIM* ODER TITEL = PFLEGEHEIM* ODER ABSTRACT = ALTENHEIM* ODER ABSTRACT = PFLEGEHEIM*		4857
**#**	FS = (ENTRY OR TRANSITIONS* OR TRANSITION OR PLACEMENT OR ADMISSION* OR ADMISSION OR DISCHARG* OR DISCHARGE OR EINZUG OR UMZUG OR ÜBERGANG OR EINTRITT OR ENTLASSUNG OR ÜBERLEITUNG)		1370
**#**	#1 UND #2		116

### Selection of sources of evidence

We used the systematic review software ‘Covidence’ (Veritas Health Innovation, Melbourne, Australia. Available at www.covidence.org) for the study selection. Titles, abstracts, and full texts were screened independently by two reviewers (out StS, TD, or RT) according to the eligibility criteria.

Disagreements between reviewers were discussed and solved by consensus. We used the updated Preferred Reporting Items for Systematic Reviews and Meta-analyses (PRISMA) flow diagram for ‘new systematic reviews which included searches of databases, registers and other sources’ [36] to document the literature search and selection process.

### Data charting process

We used an adapted version of the data charting form as recommended by Peters et al. (2020) [32] (see additional file 2). Since data charting is an iterative process, the form was further developed in the team, pre-tested with three exemplary studies and adopted. Data charting and extraction was carried out by a single researcher and double-checked by another (StS and TD). The data charting form was reviewed by two other researchers (JH and MM) to ensure accuracy.

### Synthesis of results

We narratively summarized the characteristics of the included studies. Challenges and care strategies were identified in the data by qualitative structured content analysis using MAXQDA 2020 (VERBI Software, 2020) with the TRANSCIT (TRANsition, Support, Communication, Information, and Time) model as an underlying theory [37]. Categories were developed both deductively and inductively. The TRANSCIT model was developed to improve transitional care and describe the needs of older persons and their informal caregivers during the transition from home to nursing home. The model describes three transition phases: 1) The pre-transition phase which comprises the decision-making process on the admission to a nursing home. 2) The mid-transition phase which contains the period until the relocation is completed. 3) The post-transition phase which describes the adaptation to the nursing home after the relocation [37]. Furthermore, the model describes an overall need for a partnership between persons in need of care, informal caregivers and health professionals throughout the whole transition process. It is reflected in four key components: information, time, support, and communication.

## Results

**Fig. 1:**
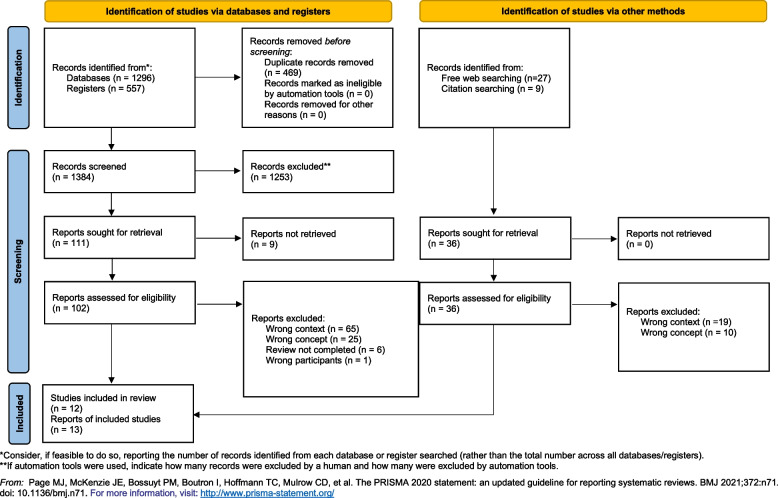
PRISMA 2020 flow diagram

**Fig. 2:**
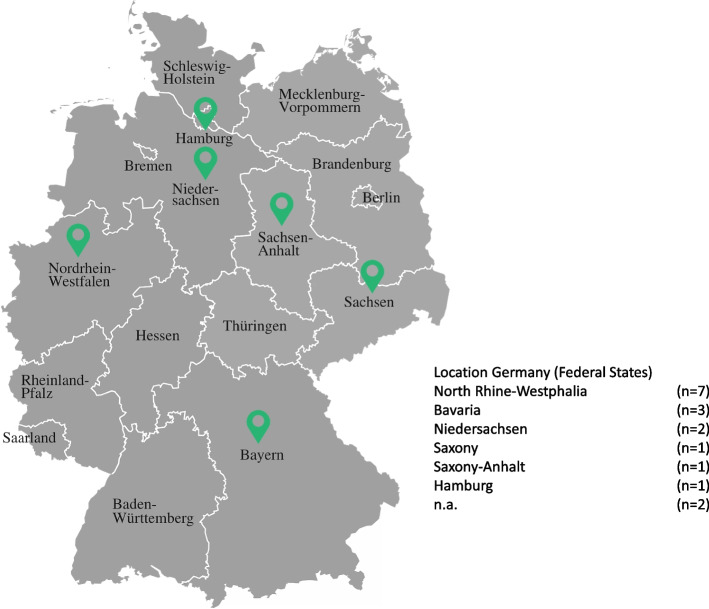
Locations of included studies in Germany (Federal states)

A total of 1,384 record titles and abstracts were screened and full-text records of 138 studies were assessed for eligibility. Finally, we included 12 studies (published in 13 reports) (Fig. 1). Most often, full texts were excluded due to wrong contextualities (wrong country, missing IMRaD structure etc.). The final 12 studies included nine journal publications, three doctoral theses (two books, one web document), and one project report. One study was published in both a journal article and a book, resulting in 12 final studies published in 13 reports. The discharging settings were home (*n* = 6) and hospitals (*n* = 4). Two studies did not report the discharging setting. The publication dates ranged from 2005 to 2020 with a median at 2014.5. The studies were conducted in different federal states of Germany, most of them in North-Rhine Westphalia (*n* = 7, Fig. 2). A total of 11 studies collected primary data, among those, five reported to have ethical clearing or votes from institutional review boards [36, 38–41], six did not provide this information [42–47]. Challenges and care strategies were extracted from 11 studies whereas one study only reported challenges. Table 3 provides an overview of the main study characteristics.

**Table 3:** Study characteristics of the studies included in the review

Authors, year	Type of evidence source	Context (Federal state of Germany; discharging/admitting setting)	Objective/Aim	Design	Data collection methods	Data analysis methods	Participants
Nguyen et al., 2018 [48]	Journal publication	North-Rhine-Westphalia Home to nursing home	To investigate experiences and views of informal caregivers and healthcare professionals regarding the transition of people with dementia to a nursing home	Secondary: qualitative cross-sectional study	Focus group interviews	Structured content analysis	Informal caregivers (of persons with dementia and one person with dementia) and interest representatives (n = 17); age: 47–90; healthcare professionals (n = 13); age: 32–62
Stephan et al., 2013 [41]	Journal publication	North-Rhine-Westphalia Home to nursing home	To investigate reasons for nursing home entry from the perspectives of informal caregivers and experiences during the first weeks after entry	Primary: qualitative explorative	Interviews	Inductive content analysis	Informal caregivers of persons with dementia, n = 114; age: 38–91 (mean: 59)
Koppitz, 2010 [38]	Journal publication and doctoral thesis (book)	Bavaria To Nursing home	To gain insight into the experiences of nursing residents in the first 3 months after nursing home entry	Primary: qualitative explorative	Interpretative phenomenology, guided interviews	Interpretative phenomenology; thematic analysis	Nursing home residents (n = 12); age: 68–93 (mean: 83) informal caregivers (n = 12); age: n.a
Hartmann et al., 2017 [36]	Journal publication	North-Rhine-Westphalia To Nursing home	To investigate which tasks informal caregivers perform in the nursing home during the first months after nursing home entry	Primary: longitudinal study	Standardised questionnaire (InterRAI, frequency and duration of nursing home visits)	Descriptive statistical analysis	Informal caregivers of persons with dementia (n = 119); age: 32–91 (mean: 60)
Schulte et al., 2017 [40]	Journal publication	Niedersachsen Hospital to nursing home	To investigate the technical and organizational feasibility, usability, usefulness and completeness of an electronic instrument (based on the German HL7 CDA standard for eNursing Summaries) for information transmission between one setting to another	Primary: cross-sectional feasibility study	Mixed methods; document analysis, standardized questionnaire (IsoMetrics), logbooks, transfer forms / reports, focus group interviews	Case analysis, qualitative content analysis, descriptive statistical analysis	*Cooperation partners: 9 inpatient* + *4 home care institutions; 1 maximum care hospital; users:* n = 26 (14 hospital nurses, 12 nurses of nursing homes); patients (n = 14); sender of transfer E-reports (n = 10, receiver of transfer E-reports (n = 9); sender of paper-based transfer reports (n = 5), receiver of paper-based transfer reports (n = 5), n = 69, institutions (provision of care transition forms); age: n.a
Reinspach & Kraus, 2006 [46]	Project report (web document)	Bavaria To nursing home	To evaluate the effectiveness, cost-efficacy, acceptance, quality of and satisfaction with the programme‚ Pflegeüberleitung’ (care transition)	Primary: longitudinal mixed methods	Mixed-methods; questionnaires, problem-centred / expert interviews; group discussions/group interviews, workshop	Descriptive statistical analysis of documents, qualitative analysis of documents, case analysis	Nursing homes (n = 40); nursing home residents (n = 7); relatives (n = 5); nurses (of nursing homes); care transition nurses (n = 40); other staff (hospital staff, social service, staff from the social services department, other experts ((n = 6)); nurses and management of nursing homes (n = 560); age: n.a
Neubert, 2016 [45]	Journal publication	n.a. (North- and South-Germany) Home to nursing home	To investigate how informal caregivers experience the waiting period until a place in a nursing home gets vacant	Primary: qualitative explorative	Guided episodic interviews	Qualitative content analysis	Informal caregivers (n = 6); age: 45–84 (one person n.a.)
Hesse & Klewer, 2013 [44]	Journal publication	Saxony Hospital to nursing home (and other facilities)	To analyse the requirements on the nursing discharge management of a general hospital from the perspective of aftercare institutions	Primary: quantitative cross-sectional	Standardized anonymous questionnaire	Descriptive statistical analysis	22 nursing homes, 1 acute hospital, (other aftercare institutions: outpatient nursing services (n = 22); assisted living facilities (n = 3); short-term care facilities (n = 7); rehabilitation facilities (n = 14)); age: n.a
Bräutigam et al., 2005 [42]	Journal publication	North-Rhine-Westphalia Hospital to nursing home (and other facilities / home)	To investigate to what extent the “Pflegeüberleitung” (care transition) contributes to the assurance of continuity of care	Primary: qualitative Primary: cross-sectional	Qualitative, participatory semi-structured Observation, documentation using a semi-structured data-gathering instrument, questions to the involved HPs after each situation	Descriptive running text, structured content analysis and discussion with professionals, interpretative evaluation (hermeneutics)	3 hospitals, 980 situations / 100 shifts (involving 4 patients per institution and the involved HPs); age: n.a
Ernst, 2019 [43]	Doctoral thesis (web document)	North-Rhine-Westphalia Home to nursing home	To investigate transitions of care- needing people with dementia from home settings to nursing homes to gain knowledge about the special needs of this group of patients and about the reasons for their transfer	Primary: qualitative explorative	Guided interviews	Qualitative content analysis	Relatives (informal caregivers) who had accompanied patients into formal nursing home settings (n = 17); age: 76–97 (mean: 86,9)
Pieper & Kolankowska, 2011 [39]	Journal publication	North-Rhine-Westphalia Hospital to nursing home (and other facilities / home)	To evaluate the status quo of transition in a major German city after standardization of procedures and implementation of standard forms to evaluate satisfaction with handling of standard forms and improvement of procedures and satisfaction of patients with the discharge process	Primary: quantitative cross-sectional	Standardized questionnaires	Inductive bivariate statistics	Nursing homes (n = 41), nursing services (n = 27), rehabilitation clinic (n = 1), GPs (n = 27); age: n.a.; hospitals (n = 13); patients (n = 634); age: mean: 62 (+ -15 years)
Zielke, 2020 [47]	Doctoral thesis (book)	North-Rhine-Westphalia; Niedersachsen; Saxony-Anhalt Home to nursing home	To investigate the nursing home transition relating to housing and the design possibilities	Primary: longitudinal qualitative	Participatory observation 4 field phases (1 in an assisted living facility), comprehensive interviews	Grounded theory	Nursing homes (n = 3); nursing home residents (n = 29); age: n.a

Most of the categories could be attributed to a specific transition phase (pre-, mid-, or post-transition phase) and to one of the four key components (time, support, communication, and information) of the TRANSCIT model. Some of the identified challenges and strategies were present in all phases of the transition. Thus, we decided to create two additional categories, ‘overarching challenges’ and ‘overaching care strategies’.

## Pre-transition phase: challenges

### Support

#### Advice & support from HPs

Two studies showed a lack of advice and support from HPs during the pre-transition phase [43, 48]. Furthermore, a lack of support and guidance from other HPs, such as hospital staff and general practitioners (GPs) or medical specialists was reported. GPs and medical specialists were hard to reach. The informal caregivers expected better psychosocial support from GPs [48] and expressed feelings of being left alone [43]. These disappointments lead to less or even no appointments with GPs and medical specialists [43]. Professional advisory or supportive offers (i.e. from long-term care insurances or other professional advice centres) were either rarely used by the informal caregivers or often regarded as ineffective and not helpful [43]. It was reported that long-term care (LTC) insurances frequently did not provide consulting and advice even though they were legally obliged to do so.

#### Financial support

In one study, informal caregivers pointed to inadequate reimbursement for nursing home costs as a barrier to nursing home placement [48].

### Communication

#### Empathy from HPs

A lack of empathy and understanding from HPs was reported. In particular, in one study it was elaborated that hospital staff and GPs did not try to appraise the feelings of the caregivers or the patients [42], showed lack of empathy for the burdensome situation and refused to engage in a dialogue [42, 43].

### Information

#### Negative public perception / experiences

It was reported in one study that previous negative experiences with nursing home care led to difficulties in the decision-making process [45]. The negative public perception and representation of nursing homes in the media may contribute to these difficulties and reinforce fears of a nursing home entry [45, 48].

### Time

#### Unprepared / sudden need for decision

Four studies reported a sudden, unprepared need for decisions [43, 46–48]. The decision for a nursing home placement is often made late or under high pressure when informal caregivers are unable to cope with the situation at home anymore. Frequently, the admission to a nursing home takes place after an acute hospital treatment and sudden deterioration of health status. Preventive and prepared decision processes are mostly lacking and informal caregivers experience sudden separation and unwanted institutionalization.

## Mid-transition phase: challenges

### Support

#### Giving up personal belongings

In one study it was highlighted that giving up personal belongings could be challenging for people in need of care in the mid-transition phase [47]. People in need of care are often unable to bring all their personal belongings into the nursing home due to limited space. It was reported that seniors needed support to establish a sense of home during this time.

#### Role change

According to three studies, both informal caregivers and older persons, who are confronted with the termination of a home care situation, may be going through a severe emotional adaptation process and role change [43, 45, 47]. Coping with this situation can vary depending on the organization of the shared time at home before the relocation, on the one hand, and the level of consensus achieved between the persons in need of care and their informal caregivers on the decision for a nursing home admission, on the other [45].

### Communication

#### Inadequate cooperation between care providers

Cooperation between the different care providers was another challenge as reported in different studies [42, 44, 46, 48]. The lack of cooperation between involved actors (people in need of nursing care, informal caregivers, HPs) and the lack of interprofessional cooperation [42] were reported to lead to fragmented and discontinued care [44, 48]. Furthermore, it was reported in one study that the interface management, i.e., written agreements on coordinated work was inadequate [46]. One study has argued that the implicit logic of the hospital hinders a perspective across systems and has to be changed fundamentally [42].

### Information

#### Information flow

The information transmission between care providers and the people in need of nursing care is often ineffective and thus appears to be a challenge in the transition process [39, 40, 42, 46]. Approaches to improve this situation (i.e. standard forms, electronic instruments) were not used on a regular basis as reported in two studies [39, 40]. Furthermore, one study revealed that the individual needs of hospitalized people in need of nursing care were poorly assessed [42].

### Time

#### Transitional care tasks

Transitional care tasks pose a high burden in terms of time to the nurses of a nursing home as stated in one study [46]. It is argued that it is hardly possible to adequately fulfil transitional care tasks on top of regular duties.

#### Waiting period

One study reported that the waiting period until nursing home admission could be challenging for informal caregivers [45]. They were confronted with different burdens – for some of them, the waiting period was too long and energy sapping, for others it was too short and an abrupt separation when a place in a nursing home was suddenly available.

## Post-transition phase: challenges

### Support

#### Staying connected

In the post-transition phase, staying connected can be a challenge for both older persons and their caregivers, according to four studies [36, 38, 41, 47]. People in need of care stated that being institutionalized had led to a decline of participation. They wished to stay included in the former environment and to participate in life outside the facility [38]. One study reported that other nursing home residents were not perceived as adequate conversation partners [47]. In some cases there was an abrupt loss of former personal contacts and leisure activities [41]. Additionally, it was reported that informal caregivers rarely participated in musical, creative or sports activities within the nursing home [41].

#### Creating own space

Two studies examined creating own space in the nursing home [38, 47]. People in need of care stated a sudden decline of privacy and it gave them feelings of humiliation [38]. Their rooms were not perceived as ‘safe’, as they were not lockable and other persons could come in anytime. Additionally, the people in need of care were often unable to rearrange their rooms to their ideas and requirements due to limited space in the nursing home [47].

### Communication

#### Mental loads & loss of autonomy

Two studies reported that older people had to cope with various mental loads and a loss of autonomy in the post-transition phase, such as being (suddenly) dependent and having to wait for help, feeling limited and immobile, as well as having to give up freedom and individual choices [38, 47]. The loss of the former everyday life, experiencing the finiteness of life, and coping with it was perceived as challenging [38]. Another study reported negative reactions after the nursing home entry. Some of the interviewed caregivers observed, that their relatives with dementia were more confused than at home (dementia symptoms increased), suffered from boredom and dissatisfaction and gave up on themselves [41].

### Information

#### Expression of habits and routines

One study discovered that older persons tended to express their needs and habits only after months of living in the nursing home. The assessment of needs and habits, however, is often perceived as concluded in the first weeks after entry [38].

### Time

#### Slow process of transition

According to two studies, the transitional process can be slow both for people in need of nursing care and for informal caregivers. While it may take months for older people to adjust to the nursing home life [38], caregivers also stated that for a long period they experienced the feelings of loss and separation [48].

#### Forced routines and decline in continuity of care

One study reported that the nursing home entry could be associated with forced routines and a decline in continuity for the older people [38]. After the nursing home entry, older people might lose their habitual performance of everyday tasks and might be forced to comply with the predefined routines of the nursing homes. Individual needs were often neglected and care was limited to non-individual basic tasks.

### Overarching challenges

#### Lack of shared decision-making

Shared decision-making, or rather the lack of it, was a challenge that was reported in different phases of the transition [42, 43, 48]. One study reported a lack of guidance in the decision-making process of the pre-transition phase (including discussions of alternatives to nursing homes) [48]. Especially, informal caregivers of persons with advanced dementia had often no contact with home care services and, therefore, no advice before the nursing home admission, because the services did not meet their care needs. Even if a home care service was present, there was a lack of adequate guidance or support [43]. Caregivers and persons in need of care were often not involved in the decision-making process, had no opportunity to discuss the decision with hospital staff and thus were confronted with paternalistic behaviour in terms of the final decision [43]. Another study reported a lack of communication and inadequate handling of crisis events in the post-transition phase. Nursing home staff involved neither the caregivers nor the GP in making decisions, such as the one for hospitalization [43].

#### Lack of evidence-based practice

Three studies have shown that some of the HPs, involved in different phases of the transition, were lacking competencies in evidence-based practice [42, 43, 46]. One study identified a lack of development of transitional care concepts and standards, including concepts for persons with dementia [46]. Another study discovered that professionalism and competencies in the transition management of the involved HPs (doctors and nurses) varied greatly[42]. Concerning evidence-based medicine, it was reported that GPs did not adhere to existing guidelines for advice and psychosocial interventions in transitional situations [43].

#### Moral conflicts & psychosocial burdens

The decision for a nursing home entry and the entry itself may go along with different psychosocial burdens and moral conflicts for the informal caregivers, as stated by three studies[43, 45, 48]. There is a high potential for conflict in the family prior to the nursing home entry [45]. Caregivers mentioned doubts and uncertainty [43, 45], as well as guilt [43, 45, 48], especially when the family blamed them for the nursing home entry or when the care-dependent person had rejected living in a nursing home earlier [43]. Telling the care-dependent person that the stay in the nursing home is not temporary also represents an ethical conflict for the caregivers [43]. Additionally mentioned were hate, despair, sadness, overload, loss of control, not being able to cope [45] and sense of duty [48]. Furthermore, ambivalent feelings, such as guilt versus relief, can be present after the entry [45].

## Pre-transition phase: care strategies

### Support

#### Familiarizing with the nursing home

Familiarizing with the nursing home was recommended by three studies as a helpful strategy to facilitate transition processes in the pre-transition phase [43, 47, 48]. Temporary stays at the nursing home prior to the nursing home entry (i.e. short-term care, day care, visits to check the room and the atmosphere) may support slow familiarization with an inpatient setting [43, 47, 48]. Positive experiences with short-term care could then further support the familiarization process [43, 48].

#### Advice & guidance from HPs

Two studies discovered that caregivers expected greater support from GPs and medical specialists regarding advice, active guidance and psychosocial interventions [43, 48]. HPs suggested that the provision of advice addressed informal caregivers’ concerns, positive aspects of the nursing home entry, limitations and financing options [48]. Moreover, initiative was demanded from nursing care insurances regarding advisory services as well as visiting consultations [43] and for more financial support [48].

### Communication

#### Respectful communication & empathy

Informal caregivers in one study stated that they expected more respectful communication, empathy and appreciation from the involved HPs (hospital staff and GPs) [43].

### Information

#### Improvement of the public perception of nursing homes

One study recommended that HPs should present a realistic picture of nursing homes to lower the informal caregivers’ fears regarding the decision for institutional care [48].

## Mid transition phase: care strategies

### Support

#### Enabling saying farewell to the home

Enabling persons with dementia to say farewell to their home after hospitalization and prior to the nursing home entry was recommended by one study to facilitate the transition [48].

### Communication

#### Improvement in cooperation between care providers

Another care-strategy in the mid-transition phase is the improvement in cooperation and collaboration between involved care providers (i.e. hospital and nursing home) [42, 44, 46, 48]. To facilitate the cooperation, different approaches were discussed in four studies, such as offering care handovers between staff from the aftercare institution and the hospital staff prior to admission, presence of known and competent contact persons on the ward, cooperative work on a specific issue/ networking (rounds to discuss problems), use of a jointly agreed standardized forms, timely provision of the medical findings [44], the development of standards for the collaboration [46], case management [48], an overall improvement of interprofessional cooperation and the rigorous reformation of the hospital system itself [42].

### Information

#### Standardization / digitalization of transitional instruments

In two studies, the standardization of transitional instruments/ forms was shown to be useful to facilitate the exchange of information between care providers [39, 44]. Another study examined the feasibility of an electronic instrument for information transmission between settings and found it to be superior to the paper-based systems [40].

### Time

#### Timing of discharge

One study suggested that the timing of the hospital discharge should be improved so that it is possible to organize care measures before the weekend [44].

## Post-transition phase: care strategies

### Support

#### Environmental design / creating own space

Three studies considered environmental design and the opportunity to create own space in the nursing home as a supporting strategy in the post-transition phase [36, 38, 47]. It was reported that there should be opportunities to take a safe walk with the residents and that lounges and rooms should be allowed to be used for private conversations to facilitate the supporting role of the informal caregivers [36]. Furthermore, it was argued that residents should have the opportunity to choose their room in the nursing home and that the rooms should offer privacy and enough space for personal belongings [47]. Another study recommended that the consideration of room and environmental design should be part of the nursing process [38].

#### Strategies to stay connected

Four studies have shown that strategies to stay connected are important in the post-transition phase [36, 38, 43, 48]. Suggestions for maintaining contacts outside the nursing home included ‘rooming-in’ and frequent visits [43, 48], together with the role of nurses as ‘participations supporters’, thus motivating the informal caregivers (or other personal contacts) to stay in contact [38] and giving them a feeling of being welcome and actively involved in the everyday life of the nursing home resident. They can also be involved in musical, creative or sports activities [36, 43].

### Communication

#### Talks & understanding

Two studies underlined the importance of understanding the situation after the nursing home entry as the fundamentals of care and the offer of talks by nursing home staff [38, 43].

### Information

#### Biography work

It was recommended by one study that family history and relationships should be considered during the transitional process [48]; and another study stated that biography work should be started right after the nursing home entry and constantly continued and adapted [38].

### Time

#### Continuity of care

One study discussed that the staff of the nursing home should be consistent and should provide individualized and tailored care in order to give the person in need of care a sense of continuity after the nursing home entry [38].

### Overarching care strategies

#### Strengthening shared decision-making

One overarching care strategy was the strengthening of shared decision-making in every phase of the transition. Two studies stated that relatives and people in need of nursing care should have the highest possible involvement directly in the whole decision-making process of the transition, regardless of where and when the decision has to be made: at home or in the hospital in the pre-/mid-transition phase or in the post-transition phase, e.g., during crisis events [42, 43]. Another study recommended that HPs should facilitate the decision-making process with information events and giving advice about the transition [48]. The opportunity to choose the nursing home ideally in a familiar environment should also be given, according to another study [47].

#### Strengthening evidence-based practice

Another overarching care strategy, which was reported by five studies, was the strengthening of competencies in evidence-based practice of the involved HPs. Suggestions for hospital nurses include the application of nursing diagnosis procedures or nursing classification systems, biography work [38], the development of transitional care concepts [46] together with discharge management training and skills development [42, 44]. It is argued that, not only the specialized transition nurses, each nurse must also be responsible for the transition as a regular task [42]. Another study suggests that the adherence of GPs and medical specialists to guidelines on advice and psychosocial interventions should be improved [43].

#### Implementation of specialized transition staff

To handle the transition tasks, specialized transitional care staff, predominantly nurses, are seen as an important support in three studies [45, 46, 48]. Responsibilities of the staff included providing advice, guidance and empowerment to persons in need of care and their informal caregivers in the decision-making process [45, 46], providing psychological and organizational support (e.g. contacting relevant health care bodies and other HPs) [45, 46, 48], facilitating biography work, and identifying the individual need for care. The responsibilities also included taking necessary measures, initiating the assessment of the statutory LTC insurance, supporting familiarization with the nursing home, optimizing transitional standards and promoting cooperation with external facilities. Besides, making contribution to day-structuring interventions for persons with dementia and other mental health problems in case of temporal space [46] and preparing informal caregivers for their new role as informal caregivers of a nursing home resident [45] were also included among the responsibilities.

## Discussion

The present scoping review summarized the findings of 12 studies focusing on the admission of older people in need of nursing care to nursing homes in Germany. From these studies, challenges as well as care strategies have been extracted and analysed by using the TRANSCIT model. Our review revealed that various challenges existed in every transitional phase for the different persons involved.

### Summary of main results

In this review*,* neglected participation and autonomy of older people in need of nursing care [36, 38, 41, 47], moral conflicts and psychosocial burdens among informal caregivers [43, 45, 48], inadequate cooperation and collaboration between care providers and lack of shared decision-making and evidence-based practice were identified as major challenges [42, 43, 45, 48]. Even though different approaches are used to enhance cooperation and continuity, standardized implementation is lacking [39, 40, 42]. The existing national expert standard has not been mentioned as an apropriate tool in the included studies as it seems to fail to improve practice. Major identified care strategies include the strengthening of shared decision-making [42, 43, 47, 48] and evidence-based practice [38, 42–44, 46], improvements in cooperation and collaboration of care providers [42, 44, 46, 48] and introducing strategies to enable participation, autonomy and continuity of care for people in need of nursing care [36, 38, 43, 47, 48]. The introduction of specialized transitional care staff to guide the whole transition process is also recommended [45, 46, 48] even though additional actions may be necessary [42].

### Research in context

Even though our review focuses on challenges and care strategies in Germany, our findings are in line with those from other countries: poor communication and care coordination across care providers [49, 50], moral conflicts and psychosocial burdens among informal caregivers and a lack of guidance and support for them during the transition [20, 51, 52], decrease of participation and autonomy of persons in need of care [15, 53] and difficulties in shared decision-making [9, 38, 52–54]. Several care strategies and interventions to address these challenges have been synthesized in international research. For example, a recent review presented different intervention components for different actors, such as education for informal caregivers and older persons, relationships/communication, improving emotional well-being, personalized care, continuity of care, support provision, and ad hoc counseling [55]However, inconsistent intervention components, results and certainty of evidence demonstrate the need for rigorous evidence-based development of interventions that address all transitional phases [55–58].

### Overall appraisal and limitations

This scoping review has several limitations. The publications were restricted to studies from Germany. Furthermore, high-level evidence such as randomized-controlled trials is missing, resulting in a lack of generalizability. The included studies showed a variety of objectives, designs, and methods and were partly non-peer reviewed No study focused on challenges as its main outcome; interventional studies are scarce and no reviews could be included. Although the process of nursing home admission is considered challenging and tends to neglect the needs of people in need of nursing care and informal caregivers, little research is available for the German health care system. Even though we did not systematically appraise the quality, many studies – across all publication types, even those with peer-review – appeared to lack methodological rigour and transparency. During the literature search, only a few studies could be found that contained comprehensive and replicable information on methods and results. Additionally, the perspective of the people in need of nursing care seems to be underrepresented, as most of the studies focus on informal caregivers and health professionals. Many studies focus on the experiences of informal caregivers of persons with dementia, but there can be other challenges from other diseases. Reported care strategies addressed important challenges; however, they were not developed and evaluated in a comprehensive and systematic way. Also, there were no recommendations addressing the unprepared and sudden decision for nursing homes, which suggested a lack of preventive approaches.

We decided to use the TRANSCIT model for the analysis. Even though the TRANSCIT model was shown to be feasible for our analysis, it focused on the needs of informal caregivers associated with admissions from home to nursing homes and not on admissions to nursing homes in general. Furthermore, we had to adapt and extend the TRANSCIT model as a scheme for our analysis which might make direct comparisons more difficult.

## Conclusions

This review shows that there is urgent need for high quality research on transitional care strategies for nursing home admissions that can be implemented into the German health care system. The most important task is to integrate the different perspectives of the involved actors into such research in a participatory way. It will help gain a comprehensive picture and develop tailored intervention programmes that address the needs of the affected individuals with consideration of local circumstances.

As the existing national expert standards appear to be insufficient, comprehensive interventions based on existing care strategies should be systematically developed, piloted and evaluated in controlled research designs in order to provide adequate support for people in need of nursing care and their informal caregivers. The introduction of specialized transition nurses seems to be a promising approach, yet it must be refined in terms of dissemination of knowledge and distribution of tasks among the whole care team. The overall awareness about admissions/transitions should be raised.

Admissions to nursing homes in Germany are associated with various challenges for different actors involved. Knowledge about these challenges and recommended care strategies addressing them can contribute to the development of comprehensive concepts in order to improve the admission and transition to nursing homes. This review is a first step in that direction.

### Supplementary Information

**Additional file 1. **The completed PRISMA-ScR checklist. 

**Additional file** **2. **The adapted data charting form.

## Data Availability

All the data are available from the corresponding author on reasonable request.

## Abbreviations

GPs: General practitioners
HPs: Health professionals
JBI: Joanna Briggs Institute
LTC: Long-term care
PRISMA: Preferred Reporting Items for Systematic Reviews and Meta-analyses
PRISMA-ScR: Preferred Reporting Items for Systematic Reviews and Meta-Analyses extension for Scoping Reviews
PRESS: Guideline for Peer Review of Electronic Search Strategies
TRANSCIT: TRANsition, Support, Communication, Information, and Time
