# Supplementary material for: Challenges and care strategies associated with the admission to nursing homes in Germany: a scoping review
Source: BMC Nurs. 2023 Jan 5;22:5. doi: 10.1186/s12912-022-01139-y (PMC9814362; doi:10.1186/s12912-022-01139-y)
Supplement: Supplementary file 2 — Additional file 2. The adapted data charting form. [file 12912_2022_1139_MOESM2_ESM.pdf]

Data charting form

	Evidence source Details and Characteristics										Details/Results extracted from source of evidence			
Nr.	Citation Details	Publication Date	Type of evidence source	Country /Region	Cont ext	Desig n	Particip ants	Data collection methods	Data analysis methods	Objectiv e/Aim	Challe nge(s)	Care strategie(s) / Recommendations	Authors conclutions	Comm ents
1														
2														
3														
4														
5														
6														
7														
8														
9														
10														
11														
12														
